# An Investigation of the Efficient–Precise Continuous Electrochemical Grinding Process of Ti–6Al–4V

**DOI:** 10.3390/ma17081729

**Published:** 2024-04-10

**Authors:** Guangbin Yang, Pingmei Ming, Shen Niu, Ge Qin, Huan Liu, Dongdong Li, Anchao Zhang

**Affiliations:** School of Mechanical and Power Engineering, Henan Polytechnic University, Jiaozuo 454003, China

**Keywords:** efficient–precise continuous pulsed electrochemical grinding, titanium alloy Ti–6Al–4V, duty cycle, pulsed voltage, surface roughness

## Abstract

Titanium alloys have many excellent characteristics, and they are widely used in aerospace, biomedicine, and precision engineering. Meanwhile, titanium alloys are difficult to machine and passivate readily. Electrochemical grinding (ECG) is an ideal technology for the efficient–precise machining of titanium alloys. In the ECG process of titanium alloys, the common approach of applying high voltage and active electrolytes to achieve high efficiency of material removal will lead to serious stray corrosion, and the time utilized for the subsequent finishing will be extended greatly. Therefore, the application of ECG in the field of high efficiency and precision machining of titanium alloys is limited. In order to address the aforementioned issues, the present study proposed an efficient–precise continuous ECG (E-P-C-ECG) process for Ti–6Al–4V applying high-pulsed voltage with an optimized duty cycle and low DC voltage in the efficient ECG stage and precise ECG stage, respectively, without changing the grinding wheel. According to the result of the passivation properties tests, the ideal electrolyte was selected. Optimization of the process parameters was implemented experimentally to improve the processing efficiency and precision of ECG of Ti–6Al–4V. Utilizing the process advantages of the proposed process, a thin-walled structure of Ti–6Al–4V was obtained with high efficiency and precision. Compared to the conventional mechanical grinding process, the compressive residual stress of the machined surface and the processing time were reduced by 90.5% and 63.3% respectively, and both the surface roughness and tool wear were obviously improved.

## 1. Introduction

Titanium alloys have the advantages of low density, high specific strength, high thermal strength, and good corrosion resistance [1,2,3,4,5,6], and are widely used in aerospace, biomedicine, and precision engineering. However, titanium alloys are classified as an extremely difficult-to-machine material owing to their several inherent properties, e.g., low thermal conductivity, high specific strength, and exceptional resistance to corrosion [7,8]. Therefore, high efficiency and precision machining of titanium alloys is a significant challenge for traditional machining methods.

**Figure 1:**
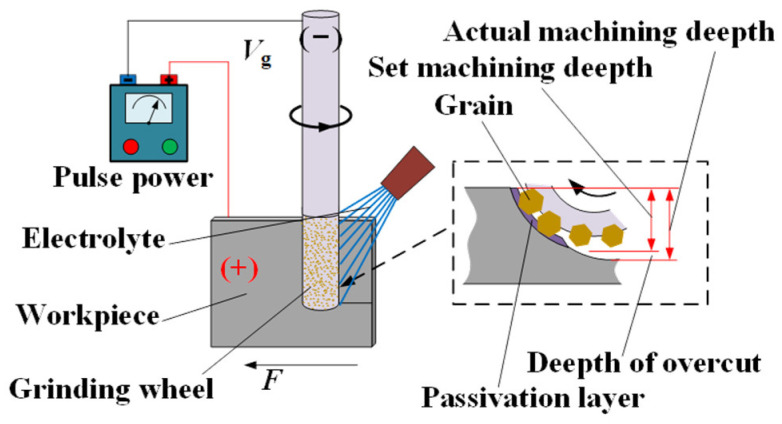
Schematic diagram of the principle of the ECG process.

Electrochemical grinding (ECG) is a hybrid machining process in which mechanical grinding and electrochemical anode dissolution act simultaneously to remove the material [9,10]. Figure 1 illustrates the principle of the ECG process. A metal-bonded grinding wheel connected to the negative pole of the power is rotated clockwise at a peripheral speed of Vg, and the workpiece connected to the positive pole of the power is simultaneously fed to the right side at a feed rate of Vw. A depth of cut, Δ, is given between the grinding wheel and the workpiece. When the electrolyte is supplied to the machining area between the grinding wheel and the workpiece, the surface material of the workpiece is electrochemically dissolved, and then a thin passive film that hinders the continued progress of the electrochemical anode dissolution is formed on the work surface in the machining area. The weakened thin passive film is easily removed by the abrasive grains, which move along with the peripheral surface of the rotating grinding wheel, and then the electrochemical anode dissolution will continue. With the circulation of electrochemical anode dissolution, a passive film is formed and removed by the abrasive grains, and the surface material of the workpiece is continuously removed until the processing requirements are met. In the process of ECG, the contribution of mechanical and electrochemical energy fields in material removal can be regulated by adjusting parameters, such as the applied voltage and the feed rate. Therefore, the process of ECG can obtain a bigger material removal rate (MRR) than mechanical grinding (MG) and be more precise than electrochemical machining (ECM) [11,12]; it is an ideal technology for the efficient–precise machining of difficult-to-machine materials that are easy to passivate.

For the last decades, considerable studies on the ECG process have been conducted. A. Hascalik [2] performed a precise ECG process on the Ti–6Al–4V surface after electrical discharge machining (EDM), applying low voltages in the range of 2–8 V, and a defect-free machined surface with a low surface roughness of 0.06 μm was obtained. In studies by J. Kozak and G. Skrabalak [13], contrast tests for different contribution rates in the material removal of electrochemical dissolution in ECG were performed by applying interelectrode voltages of 6, 8, and 10 V, respectively, and slots with a width equal to 0.3 mm were machined on a 0.2 mm thick stainless steel plate. Tehrani and Atkinson [14] studied the ECG of tool steel and stainless steel and revealed that a precise machined surface without an overcut can be obtained by applying pulsed voltage with low duty cycles of about 0.4 and 0.2 for tool steel and stainless steel, respectively.

In addition to large (150–250 mm Ø) conventional abrasive metal bond wheels applied in the above-mentioned studies, claviform metal bond grinding wheels with smaller diameters can also be used, which will further enhance the processing flexibility and application scope of the ECG process. D.T. Curtis [15] studied the ECG of a nickel-based superalloy employing grinding heads that were wheels ranging between 10 mm and 15 mm in diameter and machined a disc blade root slot structure on V-shaped pre-slotted blocks (8–9 mm thick) with a fir tree-shaped electroplated CBN grinding head. M. Nomura et al. [16,17] conducted a hybrid material removal process named Ultrasonic-assisted Electrochemical Grinding (UAECG) of Ti–6Al–4V, applying a cylindrical grinding wheel with a diameter of 1.8 mm; the grinding depth and the amplitude of the ultrasonic vibration were 3–5 μm and 4 μm, respectively, the roughness of the machined surfaces was between Ra 0.6 μm and 1 μm, and input voltages of 0–20 V were applied.

Compared with common difficult-to-machine alloys, such as stainless steel and nickel-based superalloys, titanium alloys oxidize readily. Under the action of electrochemicals, deep and compact passive film will be formed on the surface of the titanium alloy workpiece, which will hinder the continuous progress of electrochemical anode dissolution [18,19]. In the ECG process of titanium alloys, the only method to achieve high efficiency of material removal is to enhance the electrochemical anode dissolution action [20,21]. The common approach is to apply high voltage and active electrolytes to accelerate the breakage of the passive film. However, applying high voltage and active electrolytes will lead to serious stray corrosion, and the time utilized for the subsequent finishing will be extended greatly [22]. Therefore, previous studies on the ECG of titanium alloys mainly focused on the finishing process [2,3,16,17]; the application of ECG in the field of high efficiency and precision machining of titanium alloys is limited.

In the present study, an efficient–precise continuous ECG (E-P-C-ECG) process applying high-pulsed voltage with an optimized duty cycle and low DC voltage in the efficient ECG stage and precise ECG stage, respectively, was proposed, and a claviform metal bond electroplated CBN grinding wheel with a diameter of 6 mm was employed to perform the ECG process of Ti–6Al–4V. The concentration of the NaNO_3_ electrolyte for the proposed process was selected by the passivation properties test method of constant potential polarization and electrochemical impedance spectroscopy (EIS). Then, the maximum feed rate of the efficient ECG stage with different parameter combinations was tested at high electrochemical voltage, and the optimal duty cycle was selected. Finally, the machined surface obtained by the efficient ECG stage was finished at low voltage, and the flatness and surface roughness of the machined surface were improved. The residual stress and chemical composition of the machined surfaces were characterized and analyzed.

## 2. Materials and Methods

### 2.1. Material of the Workpiece

Titanium alloy Ti–6Al–4V is the first practical structural titanium alloy developed by America in 1954, and the development and application of titanium alloys have developed rapidly since then. Ti–6Al–4V workpieces are used in this work. Table 1 shows the chemical composition of Ti–6Al–4V, which has a density of 4.5 g/cm^3^.

### 2.2. Electrolyte Choosing

**Figure 2:**
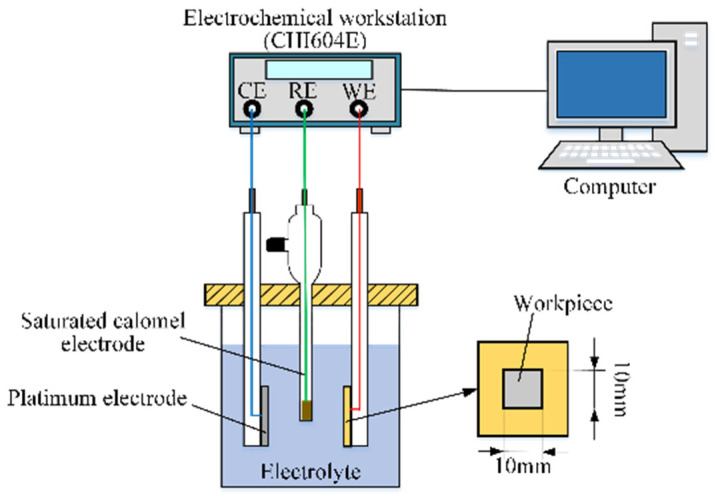
Schematic diagram of the passivation properties test system.

Passivating electrolytes, such as the NaNO_3_ aqueous solution, are generally used in the ECG process as they can effectively control the stray electrolysis and, consequently, improve the machining precision [9,23,24]. In the present study, a NaNO_3_ aqueous solution was used, and the passivation properties of the Ti–6Al–4V workpiece in the NaNO_3_ aqueous solution of different concentrations were tested by an electrochemical workstation (CHI604E, CH Instruments, Shanghai, China) and an electrochemical cell to select an ideal electrolyte. A schematic diagram of the test system is shown in Figure 2. It is a three-electrode test system, which is mainly composed of an electrochemical workstation, an electrochemical cell, and a computer. A platinum sheet (20 × 20 × 0.2 mm), a saturated calomel electrode (SCE), and a workpiece acted as the counter electrode (CE), the reference electrode (RE), and the working electrode (WE), respectively. The surface of the workpiece is parallel to the counter electrode, and only 1 cm^2^ of the surface was not covered with insulation. The electrolytes used in the test were NaNO_3_ aqueous solutions of different concentrations at a temperature of 25 °C. Before the test, the surfaces of the workpieces were ground and polished to the mirror surface and ultrasonically cleaned using deionized water.

#### 2.2.1. Constant Potential Polarization Test

**Figure 3:**
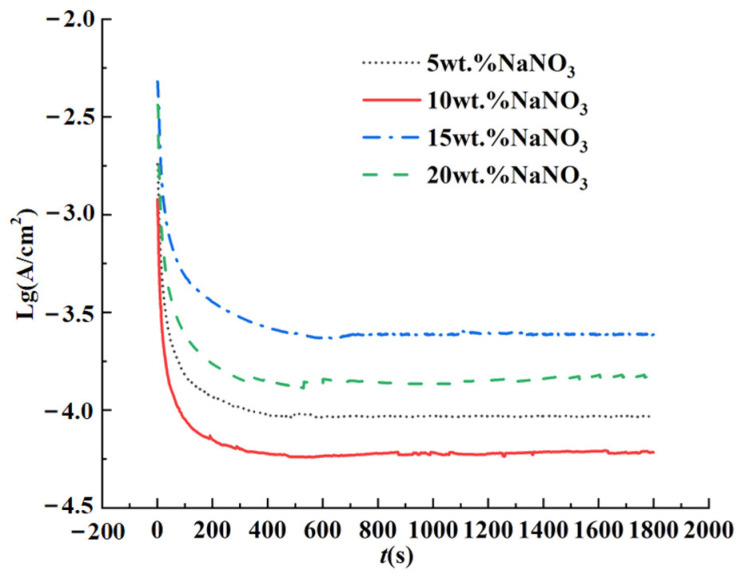
Result of the constant potential polarization test.

Liu et al. [24] tested the polarization curves for titanium alloy Ti–6Al–4V in a 10 wt.% NaNO_3_ aqueous solution. The three-electrode test system was utilized and found that the passivation potential was in the range of 0.5–11 V. In the constant potential polarization test of Ti–6Al–4V, a voltage of 8 V in the passivation potential range was selected as the passivation potential, and the constant potential polarization curves were tested in NaNO_3_ aqueous solutions of 5 wt.%, 10 wt.%, 15 wt.%, and 20 wt.%, respectively, and the tests lasted 1800 s. Figure 3 shows the test result; it is a variation of current levels as functions of time in NaNO_3_ aqueous solutions of different concentrations at a passivation potential of 8 V.

The test result reveals that the passivation current density of the specimens in different concentrations of the NaNO_3_ aqueous solution reached a stable state within 600 s, which indicates that stable passive films were formed on the surfaces of the workpieces. the passivation current density of the workpiece in the 10 wt.% NaNO_3_ aqueous solution was the smallest; therefore, the passive film formed on the titanium alloy Ti–6Al–4V workpiece in the 10 wt.% NaNO_3_ aqueous solution was the most compact.

#### 2.2.2. EIS Analyses of the Passive Film

Electrochemical impedance spectroscopy (EIS) has been already used to study the electrode system, and the EIS test performed on the surface of the workpiece can be used to analyze the properties of the passive film [25]. Before the EIS tests, the specimens were passivated at a passivation potential of 8 V in NaNO_3_ aqueous solutions of 5 wt.%, 10 wt.%, 15 wt.%, and 20 wt.%, respectively. In order to ensure that the passivation condition was the same as the actual ECG process, the passivation process was carried out on the same ECG system as the used in the experimental part of the present study, and the claviform metal bond electroplated CBN grinding wheel used in the ECG process was replaced with a metal rod cathode (6 mm Ø) to passivate the workpieces. The interelectrode gap was 50 μm, the cathode rotated at a speed of 5000 rpm, and the feed rate was kept constant at 10 mm/min. Then, the passivated workpieces were ultrasonically cleaned in deionized water.

**Figure 4:**
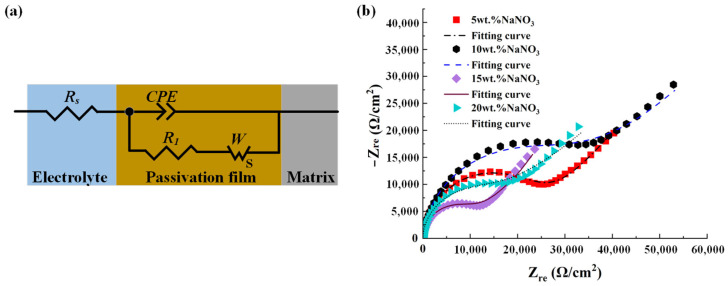
EIS analyses of the passivated Ti–6Al–4V in NaNO_3_ aqueous solutions of different concentrations: (**a**) fitting results; (**b**) the EEC model for the fitting of EIS data.

The EIS tests of the passivated workpieces were conducted by applying the test system in Figure 1. In order to ensure the accuracy of the EIS test results, the open circuit potential tests of 1800 s were first performed, and the two test stages were both conducted in NaNO_3_ aqueous solutions of the same concentration as the NaNO_3_ aqueous solution used in the passivation process. The measured open circuit potentials were used as the initial voltages in the EIS tests, and the scanning frequency was set to 0.1~100 kHz. The electrical equivalent circuit (EEC) model shown in Figure 4a was adapted to fit the data, in which R_s_ was the electrolyte resistance and R_1_ was the charge transfer resistance, and a constant phase element (CPE) was used to describe the double-layer capacitance. In order to consider the possible diffusion of the products from superficial active metals, Warburg resistance (W) was added to the circuits [26]. Figure 4b shows the fitting result of the EIS data. Nyquist plots were employed to represent the EIS data for the passivated titanium alloy Ti–6Al–4V workpieces in NaNO_3_ aqueous solutions of different concentrations. Four curves have the same trend. A semicircle at higher frequencies indicates capacitive properties of the anode interface, while a line with an angle of about 45° at lower frequencies may correspond to the diffusion process. The fitting values are listed in Table 2. Q is the capacitance of the CPE, which indicates the diffusion capabilities of ionic species within the passive film, and R_cw_ is the diffusive resistance.

The test result in Figure 4b shows that the impedance modulus (the curvature) of the semicircle capacitive arc corresponds to the 10 wt.% NaNO_3_ aqueous solution, which is the biggest, and the fitting values listed in Table 2 show that the R1 corresponds to the 10 wt.% NaNO_3_ aqueous solution, which is the largest. The test result in Figure 4b and the fitting values listed in Table 2 reveal that under the same passivation voltage (U = 8 V) and interelectrode gap (50 μm), the corrosion resistance of the passive film formed on the Ti–6Al–4V specimen, which was passivated in the 10 wt.% NaNO_3_ aqueous solution, is the highest. This is consistent with the previous test result of the constant potential polarization test.

Accordingly, considering the results of the constant potential polarization test and EIS analyses of the passive films, the 10 wt.% NaNO_3_ aqueous solution was selected as the electrolyte for the proposed E-P-C-ECG process of Ti–6Al–4V.

### 2.3. Experimental Procedure and Conditions

**Figure 5:**
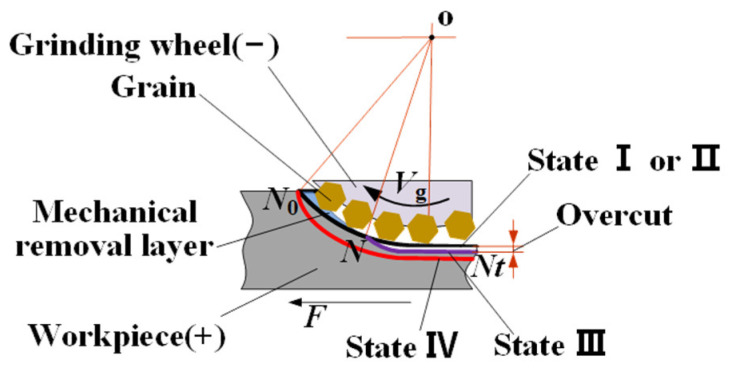
Schematic of the states in ECG.

The MRR matching between the electrochemical action and the mechanical grinding is of great importance for the efficiency and precision of ECG [15,27,28,29,30]. According to the magnitude of the applied voltage and the feed rate of the workpiece, four process states can be identified in ECG [31], as shown in Figure 5. In state I, the applied voltage (U) is in the range 0 < U < ΔE, where ΔE is the sum of the electrode potentials and over-potentials, no electrochemical dissolution is possible, and total mechanical removal occurs. In state II, the applied voltage is in the range ΔE < U, but it is insufficient to generate electrochemical anode dissolution and there will be no overcut occurring, the passive film formed on the surface of the workpiece assists the mechanical abrasive action, and total mechanical removal still occurs. As the applied voltage increases to a critical value, mechanical abrasion occurs towards the leading portion of the tool/workpiece interelectrode gap (N-N_0_), yet there is only electrochemical anode dissolution and overcut taking place directly under the center of the wheel (N-Nt), and this defines state III. While the applied voltage is further increased and the feed rate of the workpiece (V_w_) is decreased to a critical value, total electrochemical anode dissolution with no mechanical assistance occurs, and overcut takes place in the whole interelectrode gap (N-Nt); this defines state IV. Therefore, in terms of overcut, state II belongs to a precise ECG process, and state III and state IV belong to efficient ECG processes.

Previous studies [14,32] have shown that applying pulsed voltage with a low-duty cycle could reduce the overcut and improve the precision of ECG. However, the duty cycle is the ratio of pulse on time to pulse cycle time, and a duty cycle that is too small will reduce the machining efficiency and increase the wear of the grinding wheel. In order to ensure the efficiency and precision of the ECG process, the present study explored the optimal duty cycle in which a high-pulsed voltage was applied to carry out the efficient ECG stage corresponding to state III or IV, and then the precise ECG stage at a low DC voltage was carried out, which correspond to state II.

**Figure 6:**
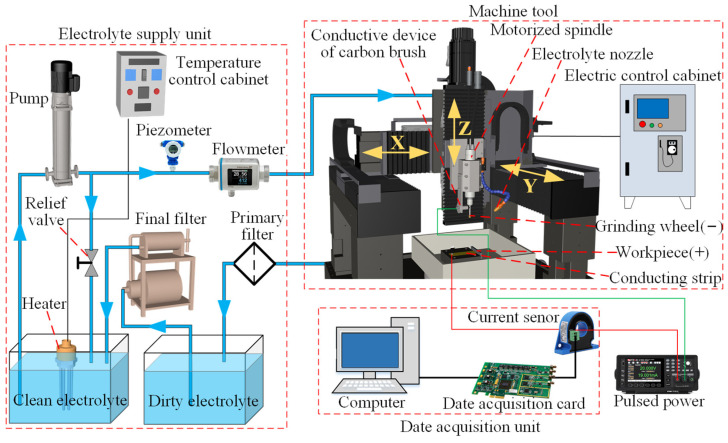
A schematic drawing of the ECG system.

Figure 6 shows a schematic drawing of the ECG system that was used to conduct the ECG process. The electrochemical grinding system consisted of an electrolyte supply unit, a machining machine, a data acquisition unit, and pulsed power. The measured repetitive positioning resolution of the machining machine was around 1 μm, which was smaller than the wheel depth of cut in the present study (see Table 3). The maximum speed of the motorized spindle (BMS-4020, NAKANISHI, Tokyo, Japan) was 20,000 rpm, and its rotation resolution was less than 1 μm. On the end of the spindle, a claviform metal bond electroplated CBN grinding wheel was installed. An electrolyte nozzle was employed to supply the electrolyte fluid into the machining area. A Ti–6Al–4V workpiece (16 × 16 × 3 mm) was held on the worktable of the machine tool via a work holder, which was made of nonconductive marble. Pulsed power was employed to induce the electrochemical reaction between the electrodes. The anode output of the pulsed power was directly connected to a conducting strip of the workpiece, and the cathode output was connected to the shank of the grinding wheel through a conductive device composed of an insulating plate, a hollow bolt, a spring, and a carbon brush.

The purpose of the experiment is to explore the optimal duty cycle of the pulsed voltage to improve the processing efficiency and precision in the efficient ECG stage of Ti–6Al–4V and conduct the precise ECG stage to obtain a precise thin-walled structure. In the efficient ECG stage, the wheel depth of cut Δ and the applied voltage U were kept constant at 100 μm and 25 V, respectively, the maximum feed rate at different duty cycles of the applied pulsed voltage was first tested, and then contrast tests of different duty cycles at the tested maximum feed rates were conducted. The optimal duty cycle of the pulsed voltage in the efficient ECG stage was selected according to the comparison result of MRR and the machined surface flatness. In the precise ECG stage, the workpiece surface machined in the efficient ECG stage using the optimized parameters was precisely machined without changing the grinding wheel, and the wheel depth of cut in the precise ECG stage was determined, according to the protruding height of the CBN abrasive grains. In order to obtain a low surface roughness in the precise ECG stage, a smaller wheel depth of cut and a higher grinding speed compared to the efficient ECG stage were applied. The specific experimental conditions and parameters are shown in Table 3.

The in-feed grinding method was adopted in this study. The initial gap between the grinding wheel and the workpiece was set to 200 μm in the feed direction, and the wheel depth of cut of the efficient and precise ECG stage was set in the grinding depth direction. The maximum feed rate at different duty cycles of the applied pulsed voltage was first tested. Then, parameter optimization tests and the E-P-C-ECG process applying the optimized parameters were conducted in turn. Each of the tests for the maximum feed rate and parameter optimization was repeated three times to ensure the reproducibility of the results. In order to calculate the MRR in the efficient ECG stage, ultrasonic cleaning and weighing of the workpieces were conducted before and after each processing. A laser confocal microscope (OLS5100-SAF, Olympus, Tokyo, Japan) was used to test the contour of the machined surfaces and measure the flatness and roughness of the surfaces. A scanning electron microscope (Merlin Compact, Zeiss, Jena, Germany) was used to observe the micro-morphology of the machined surfaces. A residual stress analyzer (Proto-lxrd, La Salle, ON, Canada) and a rotating target X-ray diffractometer (SmartLab, Yokohama, Japan) were used to test the compressive residual stress and the element composition of the machined surfaces, respectively.

## 3. Results and Discussion

### 3.1. The Efficient ECG Stage

In electrochemical grinding, the material removal process of electrochemical action and the mechanical grinding work simultaneously. A higher feed rate results in more penetration of abrasive grits into the workpiece. The mechanical grinding action will be enhanced, and the radial gap between the workpiece and the interelectrode gap will be smaller than the maximum gap that can be achieved in the electrochemical action. While the feed rate is excessively high, spark discharge, which is harmful to the grinding wheel will, be generated. Therefore, it is very important to explore the maximum feed rate for the stability of the ECG process [33].

**Figure 7:**
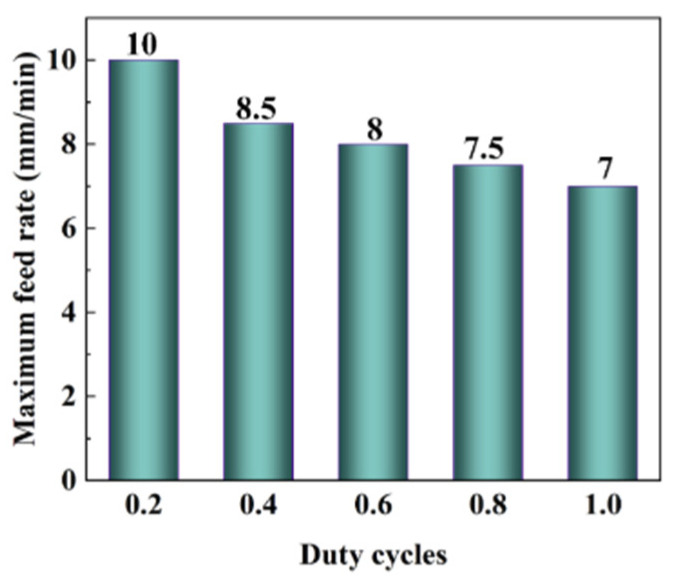
The test result of the maximum feed rate.

The maximum feed rate was defined as the maximum value of the feed rate at which no spark discharge occurs during the machining process for 5 min. Experimental parameters in Table 4 in this study were adopted in the tests to explore the maximum feed rate at different duty cycles. Each test began with a low feed rate, which was then gradually increased in increments of 0.5 mm/min until reaching the maximum feed rate [34]. The test result is shown in Figure 7. It can be seen from the result that with the increase in the duty cycle, the maximum feed rate decreases gradually; the reason is that the bigger the duty cycle is, the longer the voltage is applied, and the greater the possibility of spark discharge in the machining gap is [14], nothing except reducing the feed rate can be performed to increase the interelectrode gap to prevent spark discharge.

**Figure 8:**
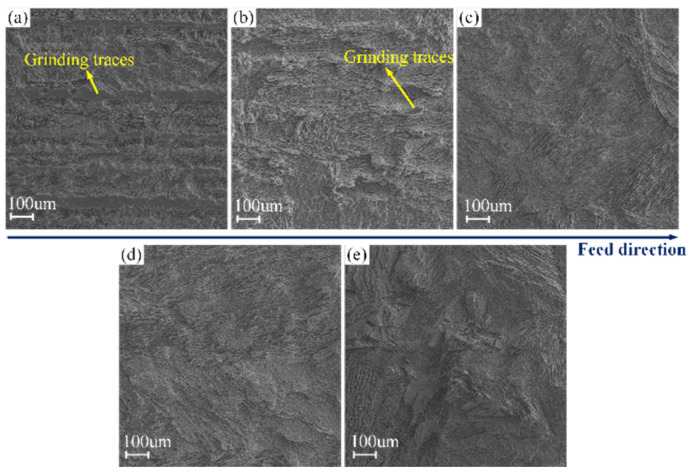
The micro-morphologies of the machined surfaces at different duty cycles observed by SEM: (**a**) 0.2; (**b**) 0.4; (**c**) 0.6; (**d**) 0.8; (**e**) 1.

The maximum feed rates of different duty cycles and the corresponding experimental parameters in Table 4 were adopted to conduct the efficient ECG stage for Ti–6Al–4V. Figure 8 shows the micro-morphologies of the machined surfaces using different duty cycles observed by the scanning electron microscope (Merlin Compact, Zeiss, Jena, Germany). It can be seen from the result that all the machined surfaces at different duty cycles are extraordinarily coarse due to serious non-uniform corrosion. A reasonable interpretation is that while Ti–6Al–4V is machined by ECM, the passive film easily generated on the surface of the workpiece is very compact. The metal matrix at the local damage will be quickly oxidized due to the oxidation of the NO_3_^−^ ion [20,35]; accordingly, it is difficult to dissolve continuously and uniformly. It can also be seen from the result that there are obvious grinding traces on the machined surface using a duty cycle of 0.2. With an increase in the duty cycle, the grinding traces gradually disappear; this phenomenon confirms that in the ECG process, applying pulsed voltage with a low duty cycle can reduce the electrochemical action and increase the mechanical grinding. Consequently, the precision of the ECG process applying a high voltage can be obviously improved by reducing the duty cycle.

**Figure 9:**
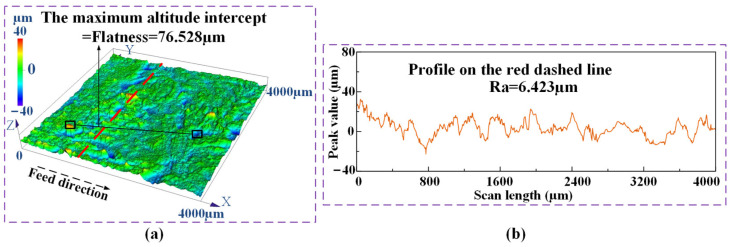
The measuring method of the flatness and roughness of the surface machined by the efficient ECG stage at a duty cycle of 0.6: (**a**) the contour; (**b**) the measured result.

In order to calculate the MRR in the efficient ECG stage, ultrasonic cleaning and weighing of the workpieces were conducted before and after each processing. The experiments at each duty cycle were repeated three times to ensure repeatability. The average value of the three calculated MRRs was taken as the experimental MRR at each duty cycle. A laser confocal microscope (OLS5100-SAF, Olympus, Tokyo, Japan) was used to test the contour of the machined surfaces and measure the flatness and roughness of the surfaces. The measuring method of the flatness and roughness is shown in Figure 9. the maximum altitude intercept, which was obtained by the analysis application of the laser confocal microscope (OLS5100-SAF, Olympus, Tokyo, Japan), was selected as the flatness of the measured surface. Red dashed lines, as shown in Figure 9a, were selected on the contour of the machined surfaces randomly to measure the roughness of the machined surfaces. The flatness and roughness were measured for the three machined surfaces at each duty cycle. the average values of the three measured results were taken as the experimental flatness and roughness of the specimens at each duty cycle. Figure 9b shows the measured flatness and roughness of the machined surface at the duty cycle of 0.6.

**Figure 10:**
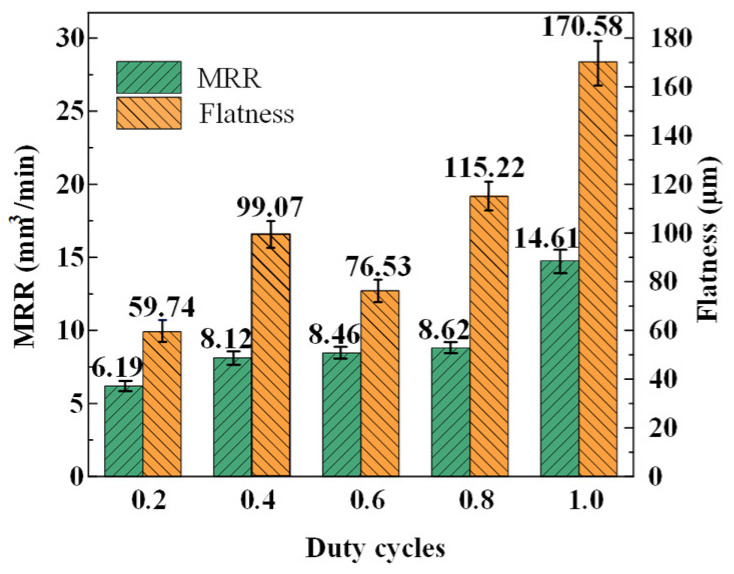
Variation in average values of flatness and MRR with duty cycles.

The average values and their error bars of flatness and MRR measure results of the machined surfaces at different duty cycles are shown in Figure 10. It can be seen from the result that with the increase in the duty cycle, the MRR shows an increasing trend; however, while the duty cycle increases from 0.4 to 0.8, the MRR increases very slowly. For flatness, as the duty cycle increases, flatness increases from 59.74 μm to 170.58 μm; however, at a duty cycle of 0.6, flatness suddenly decreases to 76.53 μm. This result indicates again that the precision of the ECG process applying a high current density can be obviously improved by reducing the duty cycle. Therefore, the matching of the electrochemical action and the mechanical grinding is better at a duty cycle of 0.6 compared to other duty cycles. In order to obtain a machined surface with a relatively small flatness to shorten the time utilized for the subsequent finishing, 0.6 was selected as the optimal duty cycle of the efficient ECG stage, and a relatively high MRR of 8.46 mm^3^/min was obtained.

According to the above results of the contrast tests and analyses, the efficient ECG stage was carried out at a duty cycle of 0.6. The rotational speed of the grinding wheel, the wheel depth of cut, the applied voltage, and the maximum feed rate were kept constant at 5000 rpm, 100 μm, 25 V, and 8 mm/min, respectively. An MRR of 8.46 mm^3^/min, and a flatness of 76.53 μm and a roughness of Ra 6.423 μm were obtained, while the optimal parameter combination was used to carry out the efficient ECG stage for Ti–6Al–4V.

### 3.2. The Precise ECG Stage

**Figure 11:**
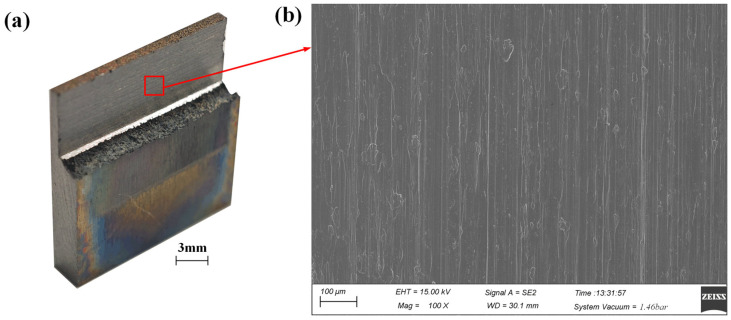
A thin-wall structure of Ti–6Al–4V machined by ECG: (**a**) the thin-wall structure; (**b**) the micro-morphologies of the machined surfaces in the red wireframe in (**a**) observed by SEM.

In the present study, a granularity of 89–104 μm was selected for the claviform metal bond electroplated CBN grinding wheel, and the average protruding height of the CBN abrasive grains was in the range of 40–50 μm. In order to obtain a low surface roughness in the precise ECG stage, a smaller wheel depth of cut, a higher grinding speed, and a larger feed rate compared to the efficient ECG stage should be applied. Previous studies have shown that the dissolution potential of Ti–6Al–4V in a 10 wt.% NaNO_3_ aqueous solution is approximately 12 V [24], and a low DC voltage that was lower than 12 V should be applied in the precise ECG stage. Optimization of the process parameters was implemented experimentally to improve the processing precision of the machined surface. Experimental parameters in Table 5 were applied to conduct the precise ECG stage for the machined surface of the efficient ECG stage because of smaller damage to the machined surface slighter tool wear and lower surface roughness according to optimized experiments, which were not described in the present study. After the efficient ECG stage was carried out by layers several times, the ECG stage was conducted by layers four times without changing the grinding wheel. A precise thin-walled structure of Ti–6Al–4V was obtained, as shown in Figure 11a, and Figure 11b shows the micro-morphologies of the machined surfaces observed by SEM.

**Figure 12:**
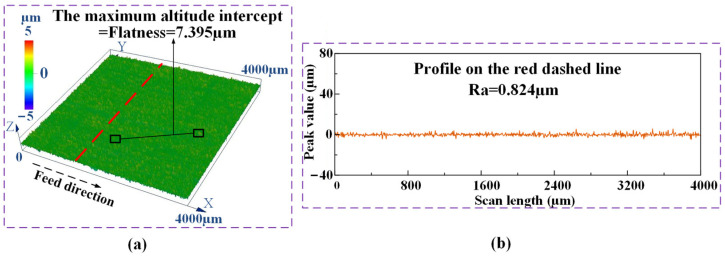
The measuring method of the flatness and roughness of the surface machined by the precise ECG stage: (**a**) the contour; (**b**) the measured result.

The same measuring method used in the efficient ECG stage was adopted to measure the flatness and roughness of the machined surfaces obtained by the precise ECG stage. Figure 12a shows the contour and the measured flatness of the machined surfaces, and Figure 12b shows the measured roughness of the machined surface. A flatness of 7.395 μm and a roughness of Ra 0.824 μm were obtained by the proposed E-P-C-ECG process for Ti–6Al–4V. This result of surface roughness is close to that obtained by M. Nomura et al. [16,17], who conducted a hybrid material removal process named Ultrasonic-assisted Electrochemical Grinding (UAECG) of Ti–6Al–4V, applying a cylindrical grinding wheel with a diameter of 1.8 mm; however, the grinding depth (5 μm) and grinding width (2.5 mm) they applied are much less than that were applied in the present study, and the MRR they obtained was much lower than that obtained in the present study.

The same mechanical machining conditions and parameters as the precise ECG stage were used to conduct the conventional mechanical grinding (MG) for the same Ti–6Al–4V specimen without applying the electrochemical machining voltage, and a thin-walled structure with the same amount of material removal as the thin-walled structure obtained by the E-P-C-ECG process was machined. A residual stress analyzer (Proto-lxrd, Canada) was used to test the compressive residual stress of the surfaces machined by the E-P-C-ECG process and the conventional mechanical grinding process. The compressive residual stress, the surface roughness, processing time, and tool wear of the thin-walled structures obtained by two processing methods were compared, as shown in Table 6. Compared to the conventional mechanical grinding process, the compressive residual stress of the E-P-C-ECG process machined surface was reduced by 90.5%, the processing time was reduced by 63.3%, and the surface roughness and tool wear were both obviously improved. A reasonable interpretation for the significant decrease in the compressive residual stress of the E-P-C-ECG machined surface compared to MG is that compressive residual stress will occur inevitably due to the existence of the cutting force; however, in both the efficient ECG stage and the precise ECG stage of the E-P-C-ECG process for the workpiece, the cutting force was significantly decreased compared to the MG process.

**Figure 13:**
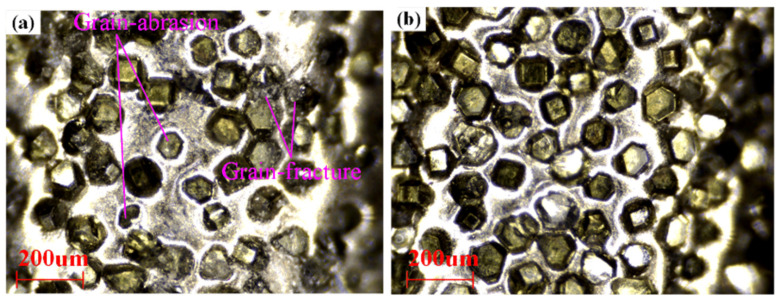
Wear of the grinding wheels that were utilized to process the thin-walled structures through two processing methods: (**a**) MG; (**b**) E-P-C-ECG.

Figure 13 shows the wear of the grinding wheels that were utilized to process the thin-walled structures by the MG and E-P-C-ECG processes, respectively. Obviously, more serious grain abrasion occurred on the grinding wheel, which was utilized to process the thin-walled structures by the MG process. This is a reasonable explanation for the larger surface roughness of the thin-walled structure, which was obtained by the MG process compared to the E-P-C-ECG process.

**Figure 14:**
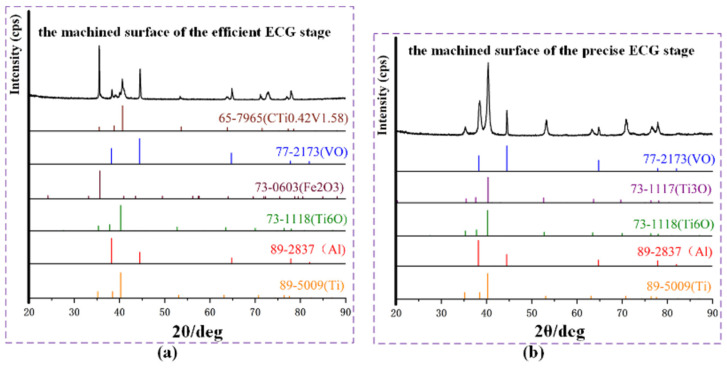
XRD patterns of Ti–6Al–4V surfaces: (**a**) surface of the efficient process of ECG; (**b**) surface of the precise process of ECG.

A rotating target X-ray diffractometer (XRD, SmartLab, Japan) was used to test the element composition of the machined surfaces. Figure 14a,b show the processing of X-ray spectra obtained from the surfaces machined by the efficient and the precise ECG stage, respectively. It can be seen in Figure 14a that the machined surface of the efficient ECG stage expatiated characteristic peaks at 35.5°, 40.7°, 53.6°, and 63.9°, which were attributed to (100), (101), (102), and (110) of CTi0.42V1.58 (PDF 65-7965), and the results flesh out that carbides of the matrix metal elements were detected on the machined surface of the efficient ECG stage. A reasonable interpretation is that the carbide skeleton in the Ti–6Al–4V alloy is difficult to dissolve under the action of electrochemicals and is easy to adhere to the machined surface; therefore, a large number of carbides will aggregate and be detected on the surface machined by the efficient ECG stage in which the electrochemical anodic dissolution is dominant. Applying a similar analysis method, the following results can be obtained. Oxides of the matrix metal elements and the matrix metal elements were detected on the machined surface of the efficient ECG stage and the precise ECG stage. The test results reveal that, in the efficient ECG stage, some residual electrolytic products of the Ti–6Al–4V in NaNO_3_ aqueous solution at high applied voltage were attached to the machined surface, and the chemical composition of the residual electrolytic products are carbides and oxides. There are simultaneously carbides, oxides, and individual elements of the matrix metal elements on the machined surface of the efficient ECG stage, which further indicate the serious non-uniform corrosion due to the extreme passivation of Ti–6Al–4V and the oxidation of the NO_3_^−^ ion. In the precise ECG stage, the electrolytic products generated in the previous process were removed layer-by-layer by the mechanical grinding of the abrasive grains. There were oxides of the matrix metal elements on the machined surface because there was no anodic dissolution except for anodic passivation in the machining area at a low applied voltage, and, consequently, there was no stray corrosion.

## 4. Conclusions

In order to explore an efficient and precise machining technique for titanium alloys with strong passivation tendencies, such as Ti–6Al–4V, the present study proposed an efficient–precise continuous ECG (E-P-C-ECG) process without changing the grinding wheel. For confirming the feasibility of the proposed technique and optimizing the process parameters, the concentration of the NaNO_3_ electrolyte for the proposed process was firstly selected by a passivation properties test, and then the effect of pulsed voltage with different duty cycles in the efficient ECG stage for Ti–6Al–4V was studied. Finally, the E-P-C-ECG experiment of the workpiece was carried out applying high-pulsed voltage with an optimized duty cycle and low DC voltage for the efficient ECG stage and precise ECG stage, respectively. The present study obtained the following results.

(1). The results of the passivation properties test of the passive film reveal that the passive film formed on the surface of the Ti–6Al–4V specimen, which was passivated in a 10 wt.% NaNO_3_ aqueous solution, was the thickest and the most compact compared to the other selected concentrations, and according to the characteristics of the ECG process, the 10 wt.% NaNO_3_ aqueous solution was selected as the ideal electrolyte.
(2). According to the experimental studies, the precision of the ECG process applying a high voltage can be obviously improved by reducing the duty cycle. In the efficient ECG stage, in order to obtain a machined surface with a relatively small flatness to short time utilized for the subsequent finishing, 0.6 was selected as the optimal duty cycle of the efficient ECG stage, and a relatively high MRR of 8.46 mm^3^/min was obtained.
(3). After the precise ECG stage, a thin-walled structure of Ti–6Al–4V was obtained with high efficiency and precision, and the roughness of the machined surface was Ra 0.824 μm. Compared to the conventional mechanical grinding process, the compressive residual stress of the machined surface and the processing time were reduced by 90.5% and 63.3%, respectively, and the surface roughness and tool wear were both obviously improved.
(4). According to the X-ray spectra obtained from the surfaces machined by the efficient and precise ECG stage, there are simultaneously carbides, oxides, and individual elements of the matrix metal elements on the machined surface of the efficient ECG stage, which indicate serious non-uniform corrosion due to the extreme passivation of the Ti–6Al–4V and the oxidation of the NO_3_^−^ ion, and the electrolytic products generated in the efficient ECG stage were thoroughly removed through the precise ECG stage.

## Figures and Tables

**Table 1:** Chemical composition of titanium alloy Ti–6Al–4V (wt.%).

Element	Ti	V	Fe	Al	H	O	N	C
Mass fraction (%)	89.335	4	0.3	6	0.015	0.2	0.05	0.1

**Table 2:** Fitting parameters of the EEC model for the passivated Ti–6Al–4V in NaNO_3_ aqueous solutions of different concentrations.

Parameters	5 wt.%	10 wt.%	15 wt.%	20 wt.%
R_s_ (Ω/cm^2^)	0.16701	0.27497	0.23459	0.17824
Q (μF/cm^2^)	7.0272 × 10^−6^	6.8198 × 10^−6^	7.7997 × 10^−6^	7.0608 × 10^−6^
n	0.92691	0.93759	0.97065	0.98734
R_1_ (Ω/cm^2^)	24,476	26,999	2609	3017
R_cw_ (Ω/cm^2^)	125,780	148,690	28,329	15,682

**Table 3:** Experimental conditions and parameters.

Items	Parameters	Value
Grinding wheel and process parameters	Type of the grains Combination	CBN Ni-Co alloy
	Granularity of the grains	89–104 μm
	Protruding height of the grains (h)	40–50 μm
	Diameter × length (ds × Ls)	6 × 10 mm
	Rotational speed of the grinding wheel (w)	5000/10,000 rpm
	Grinding speed (Vg)	94.2/188.4 m/min
	Wheel depth of cut (Δ)	100/20 μm
	Grinding width (b)	6 mm
Workpiece	Ti–6AI–4V	L16 × W16 × T3 mm
Pulsed power	Applied voltage (U)	25/4 V
	Pulse frequency (f)	5000 Hz
	Duty cycle (D)	0.2, 0.4, 0.6, 0.8, 1
Electrolyte (NaNO_3_ aqueous solution)	Concentration	10 wt.%
	Pressure	0.15 MPa
	Temperature	25 °C

**Table 4:** Experimental parameters of the efficient ECG stage.

Parameters	Value
Rotational speed of the grinding wheel (w)	5000 rpm
Wheel depth of cut (Δ)	100 μm
Applied voltage (U)	25 V
Duty cycle (D)	0.2, 0.4, 0.6, 0.8, 1

**Table 5:** Experimental parameters of the precise ECG stage.

Parameters	Value
Rotational speed of the grinding wheel (w)	10,000 rpm
Wheel depth of cut (Δ)	20 μm
Feed rate (Vw)	25 mm/min
Applied voltage (U)	4 V
Duty cycle (D)	1

**Table 6:** The compressive residual stress, surface roughness, processing time, and tool wear of the thin-walled structures obtained through two processing methods.

Parameters	Value (MG)	Value (E-P-C-ECG)
Compressive residual stresses (MPa)	556.99	52.78
Roughness of the machined surface Ra (μm)	1.907	0.824
Processing time (min)	50.56	18.56
Wear of the grinding wheel (see Figure 13)	serious	slight

## Data Availability

Data are contained within the article.
